# Interferon-γ-Inducible Protein 10 (IP-10) Kinetics after Antiretroviral Treatment Initiation in Ethiopian Adults with HIV

**DOI:** 10.1128/Spectrum.01810-21

**Published:** 2021-12-15

**Authors:** Johannes Thorman, Per Björkman, Gaetano Marrone, Taye Tolera Balcha, Fregenet Tesfaye, Tamene Abdissa, Denise Naniche, Patrik Medstrand, Anton Reepalu

**Affiliations:** a Clinical Infection Medicine, Department of Translational Medicine, Lund University, Malmö, Sweden; b Internship/Residency Unit, Skåne University Hospital, Malmö, Sweden; c Department of Infectious Diseases, Skåne University Hospital, Malmö, Sweden; d Department of Global Public Health, Karolinska Institutet, Stockholm, Sweden; e Armauer Hansen Research Institute, Addis Ababa, Ethiopia; f Adama Public Health Research and Referral Laboratory Center, Adama, Ethiopia; g Instituto de Salud Global de Barcelona (ISGlobal) Hospital Clinic, University of Barcelona, Barcelona, Spain; h Clinical Virology, Department of Translational Medicine, Lund University, Malmö, Sweden; National Institutes of Health

**Keywords:** HIV, antiretroviral therapy, viral load, tuberculosis, IP-10, resource-limited settings

## Abstract

Interferon-γ-inducible protein 10 (IP-10) has been suggested as a marker for targeted viral load (VL) monitoring during antiretroviral treatment (ART). We aimed to determine the kinetics of IP-10 during the initial year of ART, with particular regard to the impact of tuberculosis (TB) co-infection on IP-10 secretion. Longitudinal plasma IP-10 levels were quantified in 112 treatment-naive HIV-positive adults at Ethiopian health centers, through enzyme-linked immunosorbent assay (ELISA) using samples obtained before and during the initial 12 months of ART. All participants underwent bacteriological TB investigation before starting ART. In virological responders (VRs; defined as VL < 150 copies/ml with no subsequent VL ≥ 1,000 copies/ml), IP-10 kinetics were analyzed using linear regression models. Among 91/112 (81.3%) participants classified as VRs, 17 (18.7%) had concomitant TB. Median baseline IP-10 was 650 pg/ml (interquartile range [IQR], 428–1,002) in VRs. IP-10 decline was more rapid during the first month of ART (median 306 pg/ml/month) compared with later time intervals (median 7-48 pg/ml/month, *P* < 0.001 in each comparison). Although VRs with TB had higher IP-10 levels at baseline (median 1106 pg/ml [IQR, 627–1,704]), compared with individuals without TB (median 628 pg/ml [IQR, 391–885]; *P* = 0.003), the rate of IP-10 decline during ART was similar, regardless of TB-status. During the initial year of ART, IP-10 kinetics followed a biphasic pattern in VRs, with a more rapid decline in the first month of ART compared with later time intervals. Baseline IP-10 was higher in individuals with TB versus individuals without TB, but the kinetics during ART were similar.

**IMPORTANCE** To reach the goal of elimination of HIV as public health threat, access to antiretroviral treatment (ART) has to be further scaled up. To ensure viral suppression in individuals receiving ART, novel and robust systems for treatment monitoring are required. Targeting viral load monitoring to identify individuals at increased likelihood of treatment failure, using screening tools, could be an effective use of limited resources for viral load testing. Interferon-γ-inducible protein 10 (IP-10), a host inflammation mediator, has shown potential for this purpose. Here, we have investigated IP-10 kinetics in Ethiopian adults with HIV during the initial year after ART initiation. IP-10 levels decreased in parallel with viral load during ART, and prevalent tuberculosis at ART initiation did not influence IP-10 kinetics. This study shows satisfactory performance for IP-10 as a surrogate marker for viral load in persons starting ART, with no influence of concomitant tuberculosis.

## INTRODUCTION

WHO recommends universal virological monitoring for people with HIV (PWH) receiving antiretroviral treatment (ART) (1). However, access to viral load (VL) testing is limited in many areas, in particular low-income countries in sub-Saharan Africa (2). Clinical and immunological criteria, which are used for treatment monitoring if VL testing is not possible, have low predictive capacity for identification of treatment failure (3, 4). The use of algorithms or biomarkers to identify persons at increased likelihood of treatment failure, followed by VL confirmatory testing, has therefore been proposed as an alternative strategy (targeted VL testing) (5–11).

Among potential host biomarkers reflecting HIV replication, interferon-γ-inducible protein 10 (IP-10) has hitherto attracted most interest (12–14). IP-10, also known as C-X-C motif chemokine 10 (CXCL10), is a pro-inflammatory cytokine mainly secreted from monocytes and dendritic cells (15). Studies performed on ART recipients in Mozambique (16) and Ethiopia (17) have shown promising predictive capacity for identification of patients with elevated VL. However, several issues need to be resolved before IP-10 can be considered for clinical use. Patterns of IP-10 secretion may differ with regard to duration of ART, with anticipated higher levels during the initial phases of ART (18). Furthermore, several other conditions that are common in people with HIV in high-burden countries may affect IP-10 expression. In this context tuberculosis (TB), which is the leading opportunistic infection in PWH globally, is of particular importance. TB induces IP-10 secretion, and IP-10 has been proposed as a diagnostic marker for TB (19–21), as well as for monitoring of TB treatment (22). Yet, the impact of TB coinfection on IP-10 kinetics in PWH receiving ART has not been investigated.

Here, we present data on longitudinal kinetics of IP-10 expression during the initial year of ART, and the influence of TB co-infection and other patient characteristics on IP-10 kinetics. In addition, we have tested the performance of IP-10 as a screening tool for targeted VL testing during the first year after starting ART.

## RESULTS

### Participant characteristics.

**FIG 1:**
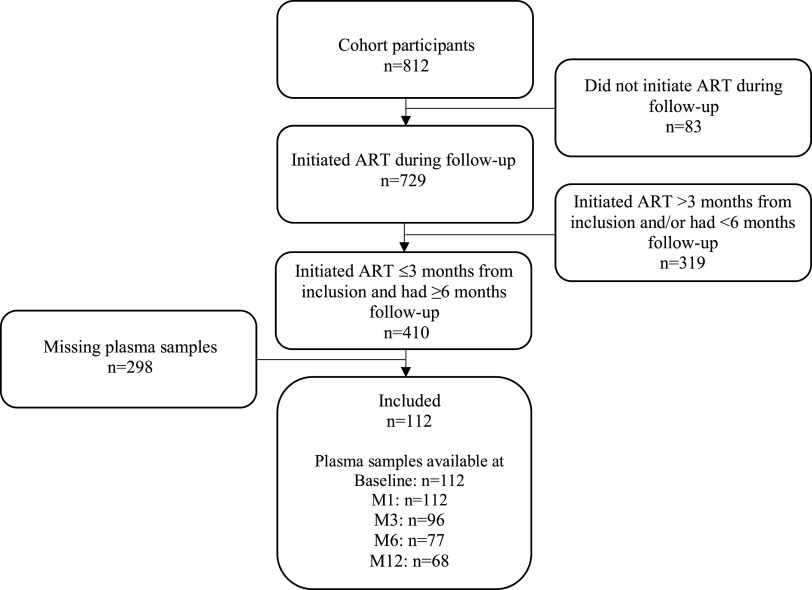
Flow chart of study participant screening process. ART, antiretroviral therapy; M, month after ART initiation.

A flow chart of selection of participants for this study is shown in Fig. 1. Stored plasma samples (for IP-10 quantification) were available from 112 of 410 individuals who met study inclusion criteria. Plasma samples from the remaining 298 persons had been destroyed due to freezer failure in the study laboratory. Characteristics were similar for individuals with and without available samples, except for lower baseline CD4 counts among included participants (Table 1). In included individuals, median age was 33 years (IQR; 28–40) and 58% were female. Median baseline VL was log_10_ 5.2 copies/ml (IQR, 4.8–5.6) and median baseline CD4 count was 156 cells/mm^3^ (IQR, 88–201). Eighteen persons (16%) were diagnosed with TB at baseline. No cases of incident TB were reported during the initial year of follow-up among those included in this study.

**TABLE 1:** Characteristics of study participants and comparison of participants with and without available plasma samples^*a*^

Characteristic	Eligible^*b*^ (*n* = 410)	Excluded due to missing plasma samples (*n* = 298)	Included (*n* = 112)	*P* value^*c*^
Age, yr	33 (28–40)	33 (28–40)	33 (28–40)	1.00
Female	235 (57.3%)	170 (57.0%)	65 (58.0%)	0.86
CD4 count, cells/mm^3^	166 (96–227)	172 (103–241)	156 (88–201)	0.03
MUAC, cm	22.0 (20.4–24.0)	22.0 (20.5–24.0)	22.0 (20.0–24.5)	0.74
Concomitant TB				
All	84 (20.5%)	66 (22.1%)	18 (16.1%)	0.17
Bacteriologically confirmed	73/84 (86.9%)	56/66 (84.8%)	17 /18 (94.4%)	0.38
Clinically diagnosed	11/84 (13.1%)	10/66 (15.2%)	1/18 (5.6%)	0.17
Baseline VL, log_10_ copies/ml	5.2 (4.7–5.6) (*n* = 189)	5.2 (4.7–5.6) (*n* = 109)	5.2 (4.8–5.6) (*n* = 112)	0.81
ART regimen				
EFV + 3TC+TDF	307 (74.9%)	217 (72.8%)	90 (80.4%)	0.12
EFV + 3TC+AZT	20 (4.9%)	11 (3.7%)	9 (8.0%)	0.07
EFV + 3TC+d4T	5 (1.2%)	5 (1.7%)	0 (0%)	0.17
NVP + 3TC+TDF	45 (11.0%)	38 (12.8%)	7 (6.3%)	0.06
NVP + 3TC+AZT	32 (7.8%)	26 (8.7%)	6 (5.4%)	0.26
NVP + 3TC+d4T	1 (0.2%)	1 (0.3%)	0 (0%)	0.54

a Data presented as median (IQR) or *n* (%). MUAC, mid-upper arm circumference; TB, tuberculosis; VL, HIV viral load; ART, antiretroviral therapy; EFV, efavirenz; 3TC, lamivudine; TDF, tenofovir disoproxil fumarate; AZT, zidovudine; d4T, stavudine; NVP, nevirapine.

b ART initiation <3 months from inclusion and ≥6 months follow-up.

c Comparing *Excluded due to missing plasma samples* with *Included* using the Mann-Whitney U test for continuous variables and Chi-square test for dichotomous variables.

Ninety-one participants (81.3%) were defined as VRs and 21 (18.7%) as virological nonresponders (VNRs). Seventeen VRs (18.7%) had TB; among these 10 (58.8%) started anti-TB treatment (ATT) before ART initiation, 4 (23.5%) started ATT within the first month after ART initiation and 3 (17.6%) started ATT at later time points during ART (Table S1 in the supplemental material).

Sixty VRs (65.9%) had complete IP-10 data up until month 6 (M6), and 41 VRs (45.1%) had complete IP-10 data up until month 12 (M12) excluding M6. These two subsets were used for longitudinal comparison of IP-10 levels and analyzed separately. Twenty-three VRs (25.3%) had complete IP-10 data at all time points and were included in both subsets.

### IP-10 levels during ART.

**FIG 2:**
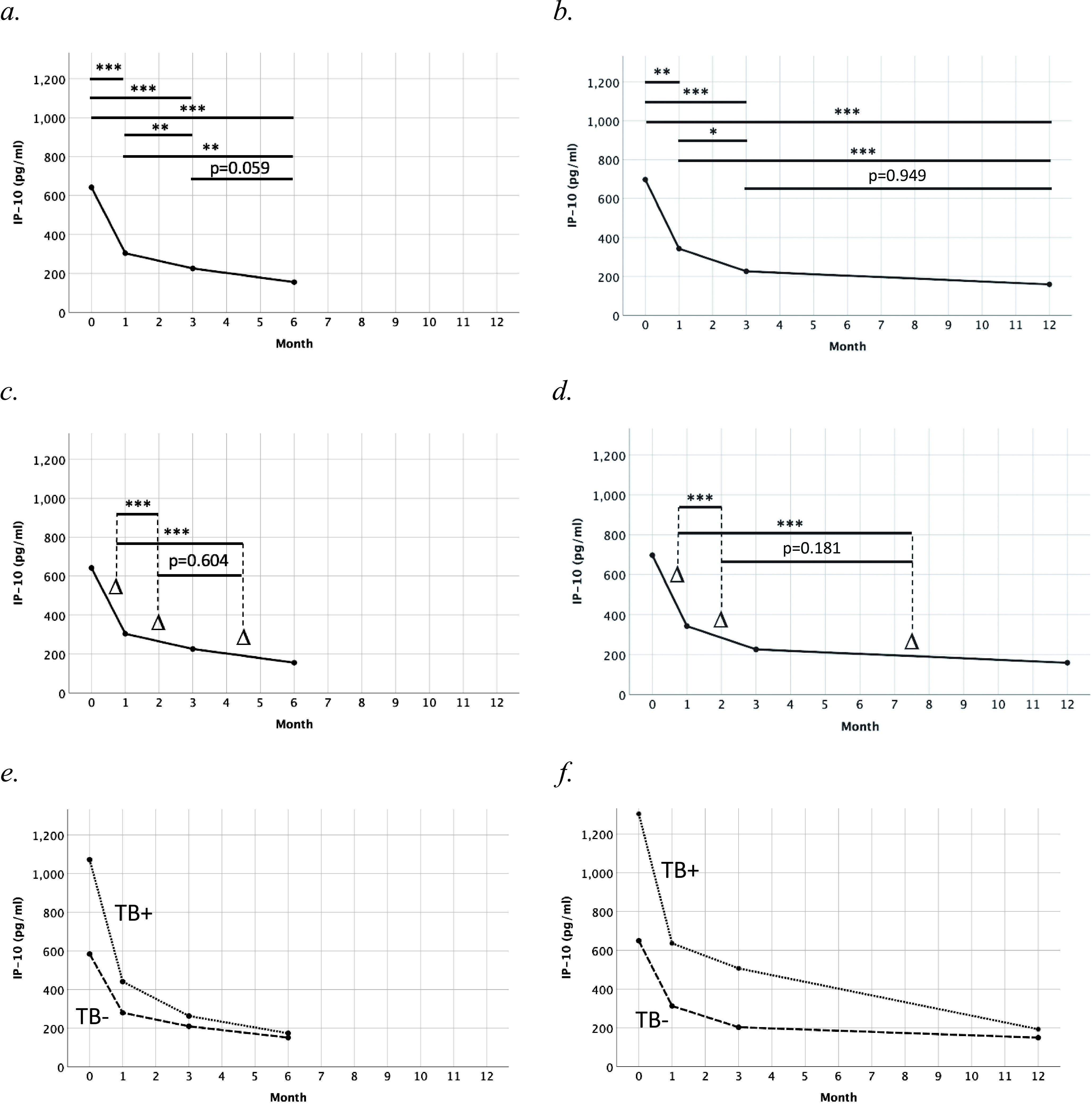
IP-10 kinetics during the initial 6–12 months of antiretroviral treatment in (a,c) VRs with IP-10 data available at B, M1, M3 and M6 (*n* = 60), (e) with (*n* = 12; dotted line) and without (*n* = 48; dash line) concomitant TB at baseline, and (b,d) VRs with IP-10 data available at B, M1, M3 and M12 (*n* = 41), (f) with (*n* = 9; dotted line) and without (*n* = 32; dash line) concomitant TB at baseline. Delta signs (Δ) represent IP-10 decline per month between two adjacent time points. *P values* correspond to pairwise comparisons from related-samples Friedman’s two-way ANOVA by ranks, adjusted by Bonferroni correction for multiple tests. IP-10, interferon-γ-inducible protein 10. *, *P* < 0.05; **, *P* < 0.01; ***, *P* < 0.001.

IP-10 levels are presented in Table 2, with median IP-10 levels for VRs with M6 and M12 data, respectively, plotted longitudinally in Fig. 2. VRs with TB had higher IP-10 levels than individuals without TB at baseline (Mann-Whitney U test, *P* = 0.003) and month 1 (M1) (*P* = 0.036) but not at month 3 (M3) (*P* = 0.072), M6 (*P* = 0.209), or M12 (*P* = 0.411).

**TABLE 2:** IP-10 levels during the initial 12 months of antiretroviral treatment in 112 HIV+ study participants^*a*^

	IP-10 (pg/ml)				
Participant sample	Baseline	M1	M3	M6	M12
All participants	634 (436–971) *n* = 112	340 (236–582) *n* = 112	226 (145–342) *n* = 96	176 (127–300) *n* = 77	189 (117–302) *n* = 68
VRs				
All	650 (428–1,002) *n* = 91	337 (235–574) *n* = 91	218 (142–339) *n* = 78	165 (126–243) *n* = 68	159 (112–262) *n* = 51
TB+	1,106 (627–1,704) *n* = 17	472 (307–737) *n* = 17	263 (189–670) *n* = 14	194 (148–333) *n* = 15	191 (132–213) *n* = 12
TB-	628 (391–885) *n* = 74	300 (215–515) *n* = 74	206 (136–317) *n* = 64	155 (125–231) *n* = 53	152 (105–269) *n* = 39
VNRs	557 (441–878) *n* = 21	406 (236–646) *n* = 21	253 (200–351) *n* = 18	319 (185–533) *n* = 9	343 (184–568) *n* = 17

a Data presented as median (IQR). IP-10, interferon-γ-inducible protein 10; M, month after ART initiation; VRs, virological responders; VNRs, virological nonresponders; TB, tuberculosis.

For individuals with M6 data, pairwise comparison of time points showed lower IP-10 levels at later time points in all comparisons except for the comparison between M3 and M6 (Fig. 2a). For individuals with M12 data, pairwise comparison of time points showed lower IP-10 levels at later time points in all comparisons except for the comparison between M3 and M12 (Fig. 2b).

IP-10 decline between time points was compared similarly. For individuals with M6 data, pairwise comparison of time intervals showed faster IP-10 decline at baseline-M1 than M1–M3 and M3–M6 respectively, but no significant difference in IP-10 decline at M1–M3 compared with M3–M6 (Fig. 2c). For individuals with M12 data, pairwise comparison of time intervals showed faster IP-10 decline at baseline-M1 than M1–M3 and M3–M12 respectively, but no significant difference in IP-10 decline at M1–M3 compared with M3–M12 (Fig. 2d).

For descriptive purposes, median IP-10 levels for VRs with and without TB respectively, were plotted for individuals with M6 and M12 data respectively (Fig. 2e and f).

### Baseline characteristics and IP-10 levels during ART.

Associations between baseline characteristics and IP-10 levels at baseline and follow-up time points were studied in VRs using linear regression models (Table 3). At baseline, concomitant TB (beta coefficient [B] 0.192, *P* < 0.01), VL (B 0.107, *P* < 0.01), and mid-upper arm circumference (MUAC; B -0.18, *P* = 0.03) were independently associated with IP-10 levels in multivariable analysis. No evidence of interaction was observed between TB, VL and MUAC (*P* > 0.5). At M1, baseline VL (B 0.137, *P* < 0.01) was the only variable independently associated with IP-10 levels in multivariable analysis. At M3 and M6, respectively, age (M3: B 0.010, *P* < 0.01; M6: B 0.008, *P* = 0.047) was the only variable independently associated with IP-10 levels in multivariable analysis. At M12, no significant association was detected in multivariable analysis.

**TABLE 3:** Linear regression of baseline factors associated with log IP-10 levels at each clinical visit in virological responders (VRs)^*a*^

	Baseline		Month 1		Month 3		Month 6		Month 12	
Variable	Uni.	Multi.	Uni.	Multi.	Uni.	Multi.	Uni.	Multi.	Uni.	Multi.
Age	0.004* (0.003)		0.004 (0.004)		0.010*** (0.004)	0.010*** (0.004)	0.008** (0.004)	0.008** (0.004)	−0.004 (0.003)	
Gender (female)	−0.044 (0.061)		0.038 (0.075)		−0.062 (0.076)		−0.057 (0.082)		0.030 (0.072)	
Baseline TB	0.257*** (0.072)	0.192*** (0.067)	0.159* (0.094)		0.181* (0.094)		0.079 (0.097)		0.065 (0.085)	
Log Baseline VL	0.145*** (0.034)	0.107*** (0.033)	0.137*** (0.044)	0.137*** (0.044)	0.078* (0.042)		0.052 (0.051)		0.062* (0.045)	
Baseline CD4 cell count	0.000* (0.000)		0.000* (0.000)		−0.001** (0.000)		0.000 (0.000)		−0.001* (0.000)	
Baseline MUAC	−0.025*** (0.009)	−0.018** (0.008)	−0.022* (0.011)		−0.016* (0.011)		−0.016 (0.013)		−0.002 (0.011)	
Intercept		2.678*** (0.268)		1.862*** (0.223)		2.023*** (0.127)				
*N*	91		91		78		68		51	
*R*^2^ (adj.)		0.250		0.089		0.080		0.044		

a Final multivariable models are shown, after stepwise removal of the least significant variable until only variables with *P* < 0.05 remained Date presented as unstandardized beta coefficient (standard error). Uni., univaritate; Multi., multivariate; TB, tuberculosis; VL, HIV viral load; MUAC, mid-upper arm circumference.

*, *P* < 0.2; **, *P* < 0.05; ***, *P* < 0.01.

Associations between baseline characteristics and IP-10 decline at specified time intervals (baseline–M1, M1–M3, M3–M6, and M6–M12) were also studied in VRs using linear regression models. Uni- and multivariable analyses were adjusted for IP-10 levels at the first time point of each analyzed time interval. No significant associations were observed, neither in uni- nor in multivariable analysis (Table S2 in the supplemental material).

### Performance of IP-10 for targeted viral load testing.

We assessed the performance of IP-10 in identifying individuals in need of targeted VL testing (defined as individuals having VL ≥1,000 copies/ml) at different follow-up time points (in all study participants, i.e., VRs and VNRs). The numbers and proportions of participants with VL ≥1,000 copies/ml at different time points were 17/112 (15.2%) at M1, 6/95 (6.3%) at M3, 5/77 (6.5%) at M6, and 15/68 (22.1%) at M12. The corresponding area under the receiver operating characteristic (ROC) curve for IP-10 to classify participants with VL ≥1000 copies/ml were 0.75 (M1), 0.68 (M3), 0.56 (M6), and 0.79 (M12). The IP-10 threshold levels for identification of VL ≥1000 copies/ml were 276.5 pg/ml (M1), 260 pg/ml (M3), 140.5 pg/ml (M6), and 186 pg/ml (M12), respectively (Table S3 in the supplemental material).

## DISCUSSION

In this study, we investigated plasma IP-10 levels in PWH during the initial year of ART. We observed a rapid IP-10 decline during the initial month after treatment initiation. Individuals with concomitant TB had significantly higher IP-10 levels before ART initiation, but both discrete IP-10 levels during ART and IP-10 decline were similar to those in persons without TB.

The marked initial IP-10 decline after starting ART corroborates findings from previous studies (23, 24). With our study design we could show that this decline mainly occurs during the initial month after ART initiation. Thereafter, IP-10 levels decline at a slower rate. This biphasic decline pattern resembles that of VL decline after ART initiation (25). Furthermore, we observed no significant difference between IP-10 levels at 3 months after ART initiation compared with 6 or 12 months, respectively, suggesting that IP-10 secretion is relatively stable after 3 months of suppressive ART. In a study conducted in Mozambique (16), median IP-10 levels for individuals with VL <150 copies/ml were markedly lower (38 versus 159 pg/ml) compared with previous data from this Ethiopian cohort (17). An obvious difference between the two studies is a longer median ART duration in the Mozambiquean study (41 months, versus 12 months in our material), which potentially could have explained the discrepancy in IP-10 levels; however, our current findings of IP-10 kinetics after 3 months of ART do not support that hypothesis.

IP-10 levels are elevated in active TB (26) and decline rapidly after initiation of ATT (27). In line with this, we found that concomitant TB was associated with higher IP-10 levels at baseline, but not at any time point after starting ART (14/17 VRs initiated ATT before or within 1 month after ART initiation). High levels of pro-inflammatory cytokines, including IP-10, are observed in TB-associated immune reconstitution inflammatory syndrome (TB-IRIS) (28–30), which is common in patients receiving concomitant ART and ATT. However, no cases of TB-IRIS occurred in the 18 persons with TB coinfection in this study. Interestingly, a pattern of IP-10 decline was found also in co-infected persons starting ART before ATT, suggesting that the inhibitory effect of blocked HIV replication on IP-10 levels is more pronounced than the stimulatory effect from ongoing TB infection (data not shown).

Previous studies have shown adequate predictive performance (sensitivity 91.5–91.7%; specificity 49.7–59.9%) for IP-10 at 12 months after ART initiation (17) and later (16) during ART. In this study, IP-10 showed similar performance at 12 months but lower performance at earlier time points during ART, albeit with wide confidence intervals (Table S3). Few individuals had VL ≥1,000 copies/ml at M3 (6.3%) and M6 (6.5%), respectively, limiting the precision of the statistical analyses on predictive performance at these time points.

With currently available technologies, and in view of the need for further scaling-up of ART delivery in resource-limited settings, there is a need to investigate alternatives to universal VL testing for ART monitoring (31). Targeting VL testing toward individuals with higher risk of viremia could potentially enable more efficient use of available resources. In particular, in settings with low frequency of virological failure, such an approach could be cost-saving with little effect on clinical outcome (8, 9). New techniques, such as GeneXpert, offer simplified alternatives to centralized HIV RNA quantification, but have certain limitations such as a low throughput capacity, relatively high cost and need for basic laboratory facilities. In this context IP-10, for which a point-of-care assay is under development, might be useful as a screening marker to identify persons at increased likelihood of having treatment failure. However, further studies are needed to determine optimal threshold levels and diagnostic performance in various settings.

This study has some limitations. Several participants from the source cohort could not be included because their stored samples had been destroyed due to freezer failure, leading to a relatively small sample size with a risk of type II error. Although the intensified TB case finding undertaken during this study suggests that the prevalence of undiagnosed TB was low in our material, several other infections that are common in PWH in sub-Saharan Africa, such as hepatitis B (32), hepatitis C (33, 34), malaria (35) and cryptosporidiosis (36) can also upregulate IP-10 expression. Systematic screening for these infections was not included in the cohort study protocol. Finally, our study participants started ART according to Ethiopian guidelines in use at the time of inclusion; hence, our findings cannot be generalized to individuals with less advanced immunosuppression at the time of starting ART.

In conclusion, IP-10 levels in patients starting ART showed a biphasic decline pattern, with a sharp decline during the initial month of ART. Persons with TB coinfection had higher IP-10 levels at baseline compared to persons without TB, but had similar kinetics during ART.

## MATERIALS AND METHODS

### Study population.

Participants were identified from a cohort study conducted 2011–2015 at five Ethiopian health centers (37, 38). For this cohort, consenting HIV-positive adults (≥18 years) who met criteria for starting ART (according to Ethiopian National ART guidelines 2012; CD4 count <350 cells/mm^3^ and/or WHO stage 4 disease) were included. Participants were followed for up to 4 years (1, 3, 6, and 12 months after ART initiation; and biannually thereafter), with venous blood sampling at each visit. Aliquots of plasma were stored at −80°C at the study laboratory (Adama Public Health Research and Referral Laboratory Center, Adama, Ethiopia). Plasma samples used in the current study had been collected at study inclusion (pre-ART); and at M1, M3, M6, and M12 after ART initiation.

At inclusion, all participants were investigated for active TB by smear microscopy, GeneXpert MTB/RIF, and liquid culture on two morning sputa and, in case of peripheral lymphadenopathy, fine-needle aspirates (39). Besides bacteriological testing, participants meeting clinical and radiological criteria (according to Ethiopian guidelines) were also diagnosed with TB. Bacteriological TB investigations were repeated during follow-up in case of clinical manifestations suggestive of active TB. Health center clinicians were responsible for starting ART and treating opportunistic infections (including TB) in accordance with national guidelines (40, 41).

For the current study, cohort participants who started ART ≤3 months after the inclusion visit, and who were followed for ≥6 months thereafter were included. In addition, available stored plasma samples from at least 3 time points (baseline; M1; and M6 or M12) were required for inclusion.

### Study definitions.

To explore IP-10 kinetics after ART initiation, and the impact of patient characteristics, we included persons showing virological response to ART (VRs). VRs were defined as individuals having VL <150 copies/ml within 6 months of ART initiation (measured at month 1, 3 and/or 6), with at least one subsequent VL <1000 copies/ml, and no VL results ≥1000 copies/ml after reaching VL <150 copies/ml during the initial 12 months of ART. Participants who did not meet these criteria were defined as VNRs.

Due to a sample availability mismatch between M6 and M12, few individuals had complete IP-10 data at all time points (baseline, M1, M3, M6 and M12). Thus, longitudinal comparison of IP-10 levels was conducted separately for individuals with complete IP-10 data up until M6 (baseline, M1, M3, M6) and individuals with complete IP-10 data up until M12 excluding M6 (baseline, M1, M3, M12), respectively. Individuals with IP-10 data at all time points were included in both analyses.

### Laboratory procedures.

HIV RNA quantification was performed in batches during the study period using Abbott Real-Time HIV-1 assay (Abbott Molecular Inc., Des Plaines, IL; lower detection limit 150 copies/ml). IP-10 levels were analyzed in duplicate on stored plasma, using the Human CXCL10/IP-10 Quantikine ELISA Kit (R&D Systems, Minneapolis, MN) according to the manufacturer’s instructions. Samples with IP-10 concentration above the assay detection limit were rerun at higher dilutions. Duplicates with coefficient of variation (CV) ≥15% were also rerun. External controls were used to measure inter-assay variability.

### Statistical analysis.

IP-10 levels at baseline and during ART were presented using medians and interquartile ranges (IQR). Plots were fitted showing IP-10 kinetics for individuals with M6 and M12 data, respectively. IP-10 levels at baseline and during follow-up were compared using related-samples Friedman’s two-way ANOVA by ranks, with pairwise comparisons adjusted by Bonferroni correction for multiple tests. IP-10 decline per month was calculated between time points (baseline–M1, M1–M3, and M3–M6 for individuals with M6 data; baseline–M1, M1–M3, and M3–M12 for individuals with M12 data) and compared longitudinally using the same test.

To study the impact of individual baseline characteristics on IP-10 levels at discrete time points, uni- and multivariable linear regression models were applied. Log transformed IP-10 levels at baseline, M1, M3, M6, and M12, respectively, were used as outcome variables in separate analyses. The following baseline characteristics were used as predictor variables: age, sex, prevalent active TB, CD4 count, log VL, and MUAC. Multivariable analysis was conducted using a backward stepwise approach in which variables with *P* < 0.2 in univariable analysis were included in the multivariable model, after which the least significant variable was removed until only variables with *P* < 0.05 remained. If two or more variables remained in the final model, we assessed whether there were interactions between the variables.

To study the impact of individual baseline characteristics on IP-10 decline a similar approach was used. Uni- and multivariable linear regression models were applied, with IP-10 decline at B-M1, M1–M3, M3–M6, and M6–M12, respectively, as outcome variables in separate analyses. The same baseline characteristics as above were used as predictor variables. These regression models were adjusted for IP-10 levels at the start of each analyzed time-interval (e.g., analysis with IP-10 decline at M1–M3 as outcome variable was adjusted for IP-10 levels at M1). Multivariable analysis was conducted using a backward stepwise approach as described above.

The performance of IP-10 for identification of individuals in need of targeted VL testing (defined as individuals having VL ≥1000 copies/ml) at different time points after ART initiation was investigated in all included participants, i.e., both VRs and VNRs, using ROC curves. Using the ROC curves, IP-10 threshold levels were determined for each time point. At these threshold levels, specificity, positive predictive value and negative predictive value with corresponding 95% confidence intervals were also calculated.

### Ethical considerations.

Ethical approval was obtained from the National Research Ethics Review Committee, Ministry of Science and Technology, Addis Abeba, Ethiopia, and the Regional Ethical Review Board, Lund University, Sweden. Written informed consent was obtained from all participants before inclusion. Impartial witnesses confirmed consent from illiterate participants.
