# Supplementary material for: Interferon-γ-Inducible Protein 10 (IP-10) Kinetics after Antiretroviral Treatment Initiation in Ethiopian Adults with HIV
Source: Microbiol Spectr. 2021 Dec 15;9(3):e01810-21. doi: 10.1128/Spectrum.01810-21 (PMC8672912; doi:10.1128/Spectrum.01810-21)
Supplement: SUPPLEMENTAL FILE 1 — Supplemental material. Download SPECTRUM01810-21_Supp_1_seq2.pdf, PDF file, 0.2 MB [file spectrum01810-21_supp_1_seq2.pdf]

Supplementary Table 1. Timing of anti-TB treatment and antiretroviral treatment initiation in 18 TB+ study participants (17 virological responders, VRs).

Treatment initiation	N	Days between ATT and ART initiation (median [IQR])
ATT before ART	10/18 (55.5%)	49 (27-63)
ART before ATT	8/18 (44.4%)	18 (10-37)

Abbreviations: ATT, anti-TB treatment; ART, antiretroviral treatment; IQR, interquartile range.

Supplementary Table 2. Linear regression of baseline variables associated with IP-10 decline between clinical visits in virological responders (VR). All analyses are adjusted for IP-10 levels at the start of each time interval. No variable met the criteria to be retained in a final multivariate model, thus no such model is presented.

	Baseline-Mo1	Mo1-Mo3	Mo3-Mo6	Mo6-Mo12
	Univariate	Univariate	Univariate	Univariate
Age	-3.9<br>(7.6)	-5.1*<br>(3.5)	-0.2<br>(1.1)	-0.04<br>(0.3)
Gender (female)	-151.5<br>(150.4)	60.6<br>(72.4)	31.5*<br>(21.5)	-13.5*<br>(7.1)
Baseline TB	179.3<br>(202.3)	-153.3*<br>(90.2)	-7.7<br>(26.9)	-10.8*<br>(7.5)
Log<br>Baseline VL	-109.8<br>(100.2)	-52.2<br>(42.5)	-1.6<br>(12.9)	-5.1<br>(4.9)
Baseline CD4<br>cell count	0.3<br>(0.7)	0.273<br>(0.306)	0.0<br>(0.1)	0.08*<br>(0.04)
Baseline MUAC	28.7<br>(24.1)	-5.1<br>(11.2)	1.7<br>(3.5)	0.4<br>(1.3)
N	91	78	60	28

Data presented as unstandardized Beta (Standard Error).

Abbreviations: TB, tuberculosis; VL, HIV viral load; MUAC, mid-upper arm circumference.

* p<0.2

Supplementary Table 3. Performance of IP-10 in identifying individuals with viral load ≥1000 copies/ml 1, 3, 6, and 12 months after starting ART.

Time-point	N	VL ≥1000 copies/ml, n (%)	AUROC (95% CI)	IP-10 threshold (pg/ml)	Se. (95% CI; %)	Sp. (95% CI; %)	PPV (95% CI; %)	NPV (95% CI; %)
M1	112	17 (15.2)	0.75 (0.65-0.86)	276.5	94.1 (71.3-99.9)	45.3 (35.0-55.8)	23.5 (19.8-27.7)	97.7 (86.4-99.7)
M3	95	6 (6.3)	0.68 (0.55-0.80)	260	83.3 (35.9-99.6)	67.4 (56.7-77.0)	14.7 (9.8-21.6)	98.4 (90.1-99.7)
M6	77	5 (6.5)	0.56 (0.29-0.83)	140.5	80.0 (28.4-99.5)	31.9 (21.4-44.0)	7.6 (4.9-11.5)	95.8 (79.4-99.3)
M12	68	15 (22.1)	0.79 (0.65-0.94)	186	86.7 (59.5-98.3)	58.5 (44.1-71.9)	37.1 (28.9-46.3)	93.9 (80.7-98.3)

Abbreviations: VL, viral load; AUROC, area under ROC curve; IP-10, interferon-gamma inducible protein 10; Se., sensitivity; Sp., specificity; PPV, positive predictive value; NPV, negative predictive value; M, month after ART initiation.

Supplementary Table 4. Viral load during the initial 12 months of ART in virological responders (VRs) and virological non-responders (VNRs).

	Baseline VL (log_{10} copies/ml)	Month 1 VL (copies/ml)			Month 3 VL (copies/ml)			Month 6 VL (copies/ml)			Month 12 VL (copies/ml)		
		<150	150-999	≥1000	<150	150-999	≥1000	<150	150-999	≥1000	<150	150-999	≥1000
VRs	5.2 (4.6-5.6)	42 (46%)	37 (41%)	12 (13%)	72 (88%)	10 (12%)	0 (0%)	87 (97%)	3 (3%)	0 (0%)	80 (96%)	3 (4%)	0 (0%)
VNRs	5.4 (4.9-5.9)	5 (24%)	11 (52%)	5 (24%)	8 (44%)	4 (22%)	6 (33%)	10 (50%)	4 (20%)	6 (30%)	2 (10%)	0 (0%)	18 (90%)

Data presented as median (IQR) or n (%).

Abbreviations: VL, HIV viral load; VRs, virological responders; VNRs, virological non-responders.

Supplementary Table 5. IP-10 kinetics in virological responders (VRs) during the initial 6-12 months of antiretroviral treatment.

VRs with available IP-10 data at baseline, M1, M3 and M6				
	IP-10 levels (pg/ml)			
	Baseline	M1	M3	M6
VRs (n=60)	642 (408-946)	304 (235-559)	226 (137-358)	156 (126-224)
VRs TB+ (n=12)	1073 (642-1823)	441 (252-673)	263 (202-606)	174 (132-339)
VRs TB- (n=48)	584 (362-851)	280 (181-518)	210 (134-327)	151 (125-224)

	IP-10 decline (pg/ml/month)		
	Baseline-M1	M1-M3	M3-M6
VRs (n=60)	301 (73-561)	42 (3-91)	21 ((-6)-38)
VRs TB+ (n=12)	622 (270-1049)	63 (1-109)	32 (17-89)
VRs TB- (n=48)	168 (61-414)	37 (3-84)	12 ((-7)-34)

VRs with available IP-10 data at baseline, M1, M3 and M12				
	IP-10 levels (pg/ml)			
	Baseline	M1	M3	M12
VRs (n=41)	697 (446-1060)	343 (243-612)	226 (144-382)	159 (114-266)
VRs TB+ (n=9)	1304 (662-2046)	636 (371-835)	507 (200-772)	193 (143-284)
VRs TB- (n=32)	650 (408-874)	313 (238-519)	203 (140-305)	150 (107-267)

	IP-10 decline (pg/ml/month)		
	Baseline-M1	M1-M3	M3-M12
VRs (n=41)	374 (113-622)	48 (3-90)	7 ((-3)-22)
VRs TB+ (n=9)	624 (316-1212)	78 ((-8)-96)	35 (6-64)
VRs TB- (n=32)	319 (74-473)	32 (1-91)	5 ((-4)-12)

Data presented as median (IQR).

Abbreviations: VRs, virological responders; IP-10, interferon-γ-inducible protein 10; M, month after ART initiation; TB, tuberculosis.
